# Physiotherapist beliefs and perspectives on virtual reality–supported rehabilitation for the assessment and management of musculoskeletal shoulder pain: a focus group study protocol

**DOI:** 10.12688/hrbopenres.13239.2

**Published:** 2022-01-25

**Authors:** Niamh Brady, Jeremy Lewis, Karen McCreesh, Beate Dejaco, Joseph G. McVeigh

**Affiliations:** 1 Discipline of Physiotherapy, University College Cork, Cork, Ireland; 2 Evolve Health, Cork, Ireland; 3 School of Health and Social Work, University of Hertfordshire, Hatfield, Hertfordshire, UK; 4 Therapy Department, Central London Community Healthcare National Health Service Trust, London, UK; 5 Department of Physical Therapy and Rehabilitation Science, Qatar University, Doha, Qatar; 6 School of Allied Health, University of Limerick, Limerick, Ireland; 7 Ageing Research Centre, Health Research Institute, University of Limerick, Limerick, Ireland; 8 Sports Medical Centre Papendal, Arnhem, The Netherlands; 9 HAN University of Applied Sciences, Nijmegen, The Netherlands

**Keywords:** Virtual Reality, Immersive, Shoulder Pain, Musculoskeletal, Rehabilitation, Qualitative

## Abstract

Shoulder pain accounts for a large proportion of musculoskeletal disorders and years lived with disability. Musculoskeletal shoulder pain is challenging to manage and while research evidence suggests that exercise should be a cornerstone of shoulder pain rehabilitation, the exact type and dosage of exercise is unclear. Adherence is a barrier to successful outcomes with exercise-based management of musculoskeletal pain, especially for those with co-morbidities, high pain levels and reported boredom associated with competing prescribed exercise. Virtual reality (VR) may offer an effective platform for rehabilitation of musculoskeletal shoulder pain. Virtual Reality has been shown to be effective for management of acute and chronic pain conditions, for delivering education around various health conditions, and for supporting rehabilitation of neurological conditions. Therefore, it is possible that VR may have a role in the delivery of exercise and education for individuals with musculoskeletal shoulder pain. VR intervention design should involve several steps and begin with establishing early acceptability from users as to the suitability of the technology in clinical practice as well as potential barriers and facilitators to using the technology successfully. This study will therefore aim to explore physiotherapists beliefs and perspectives of immersive VR as a platform for assessment and rehabilitation in patients with musculoskeletal shoulder pain. Further, this study will inform the development of a VR intervention for use in the rehabilitation of musculoskeletal shoulder pain. A series of online focus groups will be conducted with physiotherapists in Ireland using a qualitative descriptive approach to data analysis. A six-phase process of data analysis will be carried out to identify important patterns and themes within the data. The current study will be the first to explore clinician’s perspectives on the role of VR in musculoskeletal practice.

## Introduction

Musculoskeletal disorders are the major cause of years lived with disability ^1^ and epidemiological research reports that shoulder conditions are either the second, third or fourth most common musculoskeletal disorder, with a lifetime prevalence of up to 70% ^2, 3^. Paloneva *et al.* ^4^ found that in those consulting primary healthcare for shoulder disorders in Finland, the average cost of treatment per patient per year was €543, with surgical procedures accounting for the highest overall cost. Of those with shoulder pain, approximately 70% continue to experience symptoms one-year post onset ^4– 7^.

Exercise is recommended for musculoskeletal as well as general health ^8– 12^. A non-surgical management approach including exercise therapy is recommended as first line management for most individuals with musculoskeletal shoulder pain, although the best type or intensity of exercise is unknown. The literature suggests that exercise is superior to non-exercise-based therapy and specific exercise is superior to generic exercise for management of shoulder pain ^13– 16^. A recent review by Malliaras and colleagues ^17^ concluded that the evidence is unclear whether high dose is superior to low dose exercise for rotator cuff tendinopathy. Adherence to exercise-based interventions for musculoskeletal pain is often poor ^18^. Poor adherence is associated with high co-morbidities, psychological factors, pain, and boredom while performing prescribed exercise ^19– 21^.

Virtual reality (VR), known for its popularity in gaming and entertainment, has become an integral adjunctive management tool in various areas of healthcare, including pain management, neurological rehabilitation, and management of mental health disorders ^22– 24^. Many of the mechanisms underpinning the effect of VR in other populations may be applicable in managing musculoskeletal conditions. However, little research has been carried out to investigate the feasibility and efficacy of VR as an adjunctive management tool for musculoskeletal conditions. VR-based interventions for use in musculoskeletal conditions require careful design and development, feasibility testing, pilot testing and randomized controlled trials to determine efficacy ^25^.

VR-based interventions have emerged for management of both acute ^22, 26, 27^ and chronic pain ^28^. Pain can become a barrier to exercise adherence and effective pain management while exercising in VR may improve engagement ^29, 30^. Various mechanisms by which VR facilitates pain management have been proposed. These include distraction ^31^, manipulation of somatosensory input and hence perception ^32^ anxiety management ^33^ and graded exposure ^34^. Functional magnetic resonance imaging (MRI) shows that this may lead to alteration of activity in brain regions associated with the experience of pain ^35, 36^. VR has been investigated and supported as a platform for exposure-therapy in the treatment of anxiety disorders and phobias ^24, 37– 39^. Indeed, in such populations VR exposure therapy has been recommended as a more practical and attractive option than exposure therapy *in vivo* ^24^. Thomas *et al.* ^34^, demonstrated the feasibility of using a VR dodgeball game, which involved participants with low back pain bending and reaching to avoid contact with a virtual ball. Similarly, Hennessy *et al.*, ^40^ demonstrated that a VR application designed to facilitate graded exposure to everyday tasks for individuals with chronic back pain was safe and acceptable. Further research is needed to investigate the efficacy of VR exposure for individuals with musculoskeletal pain. 

Research suggests that VR may be a useful tool for educating patients and improving health literacy about various health conditions. This has been demonstrated with conditions such as atrial fibrillation ^41^ and testicular cancer ^42^ where participants’ knowledge and awareness of the conditions improved post intervention. For those with a history of atrial fibrillation, use of anti-coagulant medication increased from 57.5% prior to VR education to 70.2% and this change in behavior was maintained at 1-year follow-up ^41^. Education through VR may be useful when working with individuals with musculoskeletal conditions since management involves an active approach to rehabilitation and lifestyle change.VR has been shown as a feasible and acceptable form of rehabilitation delivery for individuals with neurological conditions ^23, 43, 44^. VR has been shown to enhance motivation and adherence to exercise programmes which require high repetitions of prescribed movements ^44^. In healthy populations, active gaming, which combines VR with physical activity, may have a role in increasing physical activity levels by making movement more enjoyable and stimulating ^30, 45^. Given the importance of therapeutic exercise and physical activity for general and musculoskeletal health, VR may have a role as a platform for exercise delivery in the management of musculoskeletal conditions.

Research investigating the efficacy of immersive VR interventions for musculoskeletal pain is emerging with promising results. A large RCT by Garcia *et al.* ^46^ demonstrated that a home-based immersive VR programme with emphasis on behavioural skills and pain education was effective for managing chronic low back pain (CLBP). Specifically, it showed that the VR programme successfully reduced pain intensity and pain-related interference with activity, mood, and stress. In this study, the VR intervention was superior to sham VR for all primary outcomes and the difference between groups was clinically meaningful. Matheve *et al.*, 2020 ^47^ used non-immersive VR to reduce pain intensity and time spent thinking about pain, in a group of individuals with CLBP during a single supervised session. For individuals with neck pain, VR has been shown to have potential as a platform for rehabilitation ^48, 49^ with improvements in outcomes lasting up to three months. A small number of studies have investigated the effectiveness of VR rehabilitation for musculoskeletal shoulder pain, specifically frozen shoulder ^50^ and subacromial pain syndrome ^51^. Both studies used non-immersive VR. Pekyavas *et al.* ^51^ conducted an RCT and found that the VR group (who completed exercise using a Nintendo Wii) showed faster improvements in the Shoulder Pain and Disability Index (SPADI) scores compared to the control group (who completed standard home exercise). Both groups showed significant improvements in pain intensity.

With musculoskeletal rehabilitation, VR interventions can take on many forms and work via various different mechanisms, depending on the needs and goals of individuals. These functions include, but are not limited to: distraction from pain; graded exposure to actvitiy; education, motivation to exercise; enjoyment; and management of anxiety. It is still uncertain whether the mechanisms underpinning the effect of VR are different for acute and chronic pain conditions ^47^. For individuals with both acute and chronic shoulder pain, many of the functions listed above may be relevant and further research to identify what individuals should benefit from specific types of VR intervention is needed.

Virtual Reality technology has the potential not only to provide pain relief and a platform for rehabilitation for people with musculoskeletal conditions, but also to provide valid and reliable assessment of individual physical performance. Gumaa *et al.* 2021 ^52^, carried out the first systematic review to evaluate the validity and reliability of VR for assessing the musculoskeletal system. This review included nine studies, four of which used immersive VR. The outcomes assessed in the studies included: range of motion (cervical spine and wrist); postural sway and balance; reaction time; velocity; and accuracy. They concluded that there is promising evidence that VR is highly reliable and valid for assessing cervical spine and wrist range of motion in asymptomatic individuals, people with neck pain and wrist fracture. To date, no study has investigated the validity or reliability of VR devices for assessing shoulder function.

VR technology is evolving rapidly, such that much of the literature demonstrating the utility of VR in various clinical settings has been carried out using non-immersive VR technology. Examples include the Nintendo Wii™ and Microsoft Kinect™ which present a computer-generated image of themselves onto a television screen. Newer “immersive” technology is based on the use of a head-mounted display unit which offers a multi-sensory experience for the user. This has implications, not only for somatosensory manipulation and hence efficacy but also for safety and feasibility. Therefore, research using up-to-date technology is indicated for exploring the utility of VR in musculoskeletal conditions. 

Birckhead *et al.* ^25^ recommend that VR-based intervention design should begin with direct input from both patient and provider end-users to optimize human-centred design, not least the acceptability of the intervention. By involving both clinicians and patients in the early stages of intervention design, it is possible to identify potential barriers and facilitators, which can then be addressed during development. Assessing acceptability early can highlight what aspects of the intervention can be modified to increase acceptability and thus participation ^53^.

### Study aims

This study aims to explore physiotherapists’ beliefs and perspectives of immersive VR as a platform for assessment and rehabilitation in patients with musculoskeletal shoulder pain and to identify potential barriers and facilitators to using VR in a musculoskeletal setting. We will encourage an open discussion and will invite participants to think about various shoulder conditions and directions for VR intervention and share ideas on how VR may be used in a clinical setting. These ideas may be influenced by participants’ clinical experience or indeed their VR experience. Further, this study will inform the development of a VR intervention for the rehabilitation of musculoskeletal shoulder pain. A future study, including patients will be carried out to further contribute to our understanding of the role of VR in the management of musculoskeletal shoulder pain and the development of said intervention.

## Protocol

### Design

This focus group study which is part of a larger mixed-methods study will use a qualitative descriptive design to explore physiotherapists’ beliefs and perspectives about using immersive VR in the assessment and rehabilitation of individuals with shoulder pain. Qualitative description was chosen for this research question as it aims to provide a straight description and detailed summary of the phenomenon of interest using participants’ own language ^54^. It therefore involves staying close to the data, through low-inference interpretation during data analysis ^55^. This qualitative approach is appropriate for this research question as it provides preliminary insight into a novel clinical intervention. Qualitative description allows for flexibility in methods of data collection and analysis ^55^, resulting in information and insight gained that is both broad and rich. Colorafi and Evans ^56^ describe qualitative description as an “excellent choice for the healthcare environments designer, practitioner or health sciences researcher because it provides rich descriptive content from the subject’s perspective”.

### Methods

A series of online focus group interviews will include physiotherapists working in Ireland. Focus groups will be conducted to explore not only what participants think about VR use in clinical practice but why they hold such beliefs. Focus groups allow for the emergence of important themes that may be overlooked in individual interviews with a more structured question schedule ^57^. They also facilitate the exploration of shared and differing views between participants. Focus groups have also been identified as an appropriate method for informing product or intervention development ^58^. Research suggests that three to four focus groups, each including eight participants is sufficient to identify important themes ^59^. This study will aim to conduct a minimum of three interviews, each including six to ten participants. However, additional focus groups will be carried out as necessary until data saturation is reached. Data collection and analysis will occur concurrently so that it will be possible to begin to identify and understand themes early on. A reflexive thematic analysis approach will be adopted. This approach is fluid in nature, allowing for coding and theme generation to happen organically and interchangeably ^60^. The research team will then decide on when coding and theme generation is complete based on the nature of the data and our research goals.

### Participants

In order to achieve study objectives, a minimum of three focus groups including between six and ten participants will be carried out. Literature recommends such numbers ensure that important themes are identified ^59^ but it is impossible to be certain how many interviews are required until data has been collected and anakysed ^60^ Krueger and Casey ^58^ suggest that larger numbers (8 participants) are appropriate when the study is designed to pilot-test new ideas and when the participants do not have a lot of background knowledge about the topic. The research team will determine whether data saturation is reached on completion of the third focus group. Purposive sampling will be used to recruit physiotherapists who work with individuals with musculoskeletal shoulder pain on a regular basis (minimum 10% total caseload). Representation will be sought from physiotherapists with varying levels of clinical experience and from a range of clinical work environments. Physiotherapists with variation in socio-demographic working environments and across public and private sectors will be recruited, as well as physiotherapists with and without previous experience of using immersive VR in any capacity (clinical or entertainment). Participants will also be recruited to represent a diverse age range and gender balance.

### Recruitment

Participants will be recruited from acute hospitals, community hospitals, primary care centres and private practices in Ireland. A study information sheet ^61^ will be sent to individual physiotherapy departments and private practices. Regulating bodies, including the Irish Society of Chartered Physiotherapists (ISCP), and relevant clinical interest groups including the Irish Shoulder and Elbow Research Society (ISERS) will be contacted and requested to distribute study information to members via email. The study will also be promoted on social media platforms; Facebook™, Instagram™ and Twitter™. The information sheet will outline the study background, aims and design. If physiotherapists are interested in participating or wish to receive further information on the study, they may contact the Primary Investigator (PI), Niamh Brady by telephone or email. Participants will be sent a consent form with a participant information sheet. Due to current coronavirus disease 2019 (COVID-19) restrictions, informed consent will be gained remotely once physiotherapists return a consent form with electronic or typed signature via email, indicating that they wish to participate. The PI will then speak to potential participants by telephone to assess appropriateness for inclusion in the study. Physiotherapists should meet the following inclusion criteria:

- Have a minimum one-year clinical experience with a minimum six months’ experience working with musculoskeletal pain conditions. Shoulder pain presentations should account for minimum 10% of their caseload.
- Willing to attend online focus group interview and consent to being video recorded.
- Being able to converse in English.

Physiotherapists will be excluded if they:

- Report suffering from severe motion-sickness
- Have a history of seizures
- Have a history of severe vertigo or vestibular impairment

Those physiotherapists who meet the inclusion criteria will be asked further questions to provide demographic information: gender, age, number of years clinical experience, current clinical setting, geographical location and previous experience of using immersive VR. Physiotherapists will then be invited to attend a focus group with date and time specified. Potential participants will be informed that they may opt out of the study at any time.

### Procedure

***Immersive VR Experience.*** For those who have not previously used VR, they will be provided with an Oculus Quest or Oculus Quest 2 VR headset for use at home prior to participation in the focus group interview. The headset will be delivered within six months prior to completion of the focus group interview. Participants may use the VR headset for up to one-week and will be advised to use the headset at least three times for a maximum of 20 minutes each time. To support their VR experience, participants will be shown an informative video, delivered via email, demonstrating how to safely use VR. They will be instructed to discontinue use if they experience adverse effects such as motion sickness and to inform the research team.

Participants will be invited to explore Oculus Quest’s native First Steps tutorial ( https://www.oculus.com/experiences/quest/3675568169182204?ranking_trace=0_3675568169182204_QUESTSEARCH_28b8d286-6206-4581-8c2c-c876d55e6943) which guides the user on how to safely set up the VR environment and how to use the hand controllers for manipulating objects in the virtual world. In addition, participants will have the opportunity to experience the demonstration activities which are available on Oculus Quest including First Steps, a popular VR rhythm game called Beat Saber ^TM^ ( https://www.oculus.com/experiences/quest/1758986534231171?ranking_trace=0_1758986534231171_QUESTSEARCH_d0b1e295-5201-4bde-a1cb-8d2440c61b2f), and a sports game called Sports Scramble ^TM^ ( https://www.oculus.com/experiences/quest/2131072803612066?ranking_trace=0_2131072803612066_QUESTSEARCH_009ba037-c88a-4b42-9363-441abbdac775). Participants will therefore have had the opportunity to experience immersive and interactive VR. They will have used their virtual hands to lift and throw objects, play table tennis, punch a virtual opponent in a boxing match, dance and explore a range of virtual worlds. Therefore, the VR experience will involve movement, concentration, and play.

***COVID-19 precautions.*** In situations where physiotherapists are provided with headsets, these will be delivered in a cardboard box by post or in person, depending on geographical location and level of restrictions in place at that time. Prior to delivery, each participant will be contacted by telephone and asked a series of questions:

- Have you been diagnosed with COVID-19 or have you been in contact with someone who has been diagnosed with COVID-19 during that last two weeks?
- Are you experiencing COVID-19 symptoms: a fever, a cough, shortness of breath, change to sense of smell or taste?
- Have you travelled overseas in the last two weeks?

If a participant answers “Yes” to any of the questions above, they will not be provided with a headset.

Participants will be instructed to clean the headset with disinfectant on receiving and before returning the headset (by post or in person). The postal box will include a pre-paid stamp so that participants have the option of returning the headset by post. Once the headset is returned, the headset will be cleaned again using disinfectant and stored in a locked cupboard for a minimum of four days, to further minimize infection risk. The PI (NB) will be responsible for distribution and collection of headsets in Ireland.

***Focus group interview.*** Participants will be invited to participate in an online focus group interview, lasting approximately 60–90 minutes. Focus groups will take place between two weeks and six months of participants having used the technology. The focus group interviews will take place over a 1-year period (March 2021– March 2022 ) With permission from participants, focus group interviews will be video recorded. Two members of the research team will be present to facilitate the focus group interview. The PI (NB) will use a semi-structured question schedule (see Extended Data) to guide the interview while an additional member of the research team (BD) will take field notes and ensure that video recording is in progress. Physiotherapists with prior relationship to the research team (colleagues, students, family members, friends) will not be invited to attend.

The semi-structured nature of the question schedule ^61^ will give the PI flexibility to adapt questions and expand on new ideas as they arise ^55, 62^. The interview questions will explore physiotherapists perspectives on how feasible a VR intervention may be in clinical practice and anticipated barriers as well as facilitators to the use of such technology. Interview questions will be piloted with two physiotherapists not included in the study beforehand to check for comprehensibility and clarity. To encourage maximum engagement among participants, various strategies will be employed to make the interviews as informal and inclusive as possible. Participants will be encouraged to converse with one another and to contribute to all sections of discussion if they feel comfortable to do so. Participants will be reminded that there are no correct or incorrect answers and that they should feel comfortable to agree or disagree with fellow participants ideas. Participants will be encouraged to converse with researchers and each other on a first name basis. It will be suggested that participants feel free to take a break at any time and to bring along tea, coffee, or snack as they wish.

***Reflexive practice.*** Research team members will participate in a reflexive practice, prior to data collection and following each focus group interview. A refection diary ^61^ will be used to document individual researchers own relationship to the research topic and the participants as well as initial thoughts regarding codes and themes. This will help to enhance quality by identifying any potential biases that may influence data collection or analysis ^63^. In our case, all members of the research team are physiotherapists. This can act as a facilitator when communicating with participants of the same profession. On the other hand, research team members must be conscious not to project their own perspectives as physiotherapists onto the participants in the group, or indeed during data analysis.

### Study setting

The focus group interviews will be conducted at University College Cork, online via Microsoft Teams.

### Ethical approval

Ethical approval has been gained from UCC Social Research Ethics Committee prior to recruitment of physiotherapists.

### Data collection and management

Prior to commencement of the focus group, basic demographic information will be collected including gender, age, number of years’ clinical experience, current clinical setting, geographical location and previous experience of using immersive VR, whether for clinical or entertainment purposes. This information will have been collected by telephone after initial screening. All demographic information will be stored in a research folder within UCC Microsoft Teams. This folder will be accessible only to members of the research team.

Further data collection will be in the form of focus group interviews. Interviews will be conducted via Microsoft Teams and will be video recorded using Microsoft Team’s native recording function. Permission for video recording will occur at two time-points; via completion of the consent form with attached information sheet which outlines the interview and recording process, and at the start of the focus group interview, before commencing the recording function. On completion of the focus group interview, the built-in recording function in Microsoft Teams stores the video recording directly to UCC One Drive which is secure and private. The recordings will then be transcribed into text by the PI (NB) and the transcription too will be saved in Microsoft Teams in Microsoft word format. The original video recording will then be deleted.

On completion of the focus group interview, participants will be informed that they have two weeks to opt out of the study. If they choose to do so, all information that they provide will be deleted and removed from analysis. A summary of each focus group interview, identifying the main points of discussion will be sent to each participant for data verification. Participants will have one further week to ask queries and change or remove data if they wish. Member checking helps to ensure trustworthiness of data ^64^. Following this process, all identifying information will be removed from the transcripts. Participants will be reassured that nothing will be published that has not been anonymized. Access to recordings, transcripts and data analysis on Microsoft Teams will be granted to members of the research team only (NB, JMcV, JL, KMcC, BD). A final check to ensure that no identifiable data exists on the research team’s equipment will be carried out. These steps are taken in compliance with the general data protection regulation (GDPR) and UCC’s data protection policy. All data will be stored for 10 years in line with the FAIR data principles.

### Thematic analysis

The transcriptions of each interview will be analyzed by two members of the research team (NB and BD). Where there is uncertainty or disagreement, a third member of the research team will be asked to contribute to the data analysis. Data collection and analysis will happen concurrently. Analysis will begin directly after each focus group is completed. A six-phase process of thematic analysis will be carried out to identify “patterns or themes within data,” ( Table 1) ^65^. We will also be guided by a framework described by Nowell *et al.* ^64^ to ensure trustworthiness in thematic analysis (). Guided by this framework, each phase of data analysis will involve various means of establishing trustworthiness, such as: prolonged engagement with data; data triangulation; peer debriefing; reflexive journaling; researcher triangulation; member checking and a clear description of the audit trail. This framework aims to achieve trustworthiness as described by Lincoln and Guba ^66^ as credibility, transferability, dependability, and confirmability ^66^.

**Table 1.** Thematic Analysis Process, Adapted from Braun and Clarke, 2012.

Phase	Tasks
Phase 1	Familiarisation with the data, including the transcription of video interviews into written text, reading and re-reading of the data, and capturing any initial ideas.
Phase 2	Creation of initial codes, a code being an identifier for some feature of the data that may be of interest and collecting the data that is relevant to those codes.
Phase 3	Analysing and sorting these codes into broader themes and collating the data associated with those codes within these themes.
Phase 4	Reviewing and refining of themes, such as discarding themes without enough data or combining themes.
Phase 5	Further refinement of themes and writing a detailed analysis of each theme.
Phase 6	Producing the final report from the detailed analysis of the themes.

Once recordings have been transcribed, transcripts will be checked against recordings for confirmation. Data will then be anonymized. Researchers will take time to independently familiarize themselves with the data. Following each individual focus group, peer debriefing will occur to members of the team not involved in data collection. Researchers responsible for data collection and analysis (NB and BD) will read and annotate a sample of transcripts independently and after discussion, agree on a broad initial coding framework which will be applied to all transcripts using NVivo software. This software facilitates data storage, organization, and comparison within and between transcripts. Themes will be derived following a rigorous process of coding, categorizing, discussion and reflection ^65, 67^. A mind-map diagram will be created to make sense of theme connections. A detailed audit trail will record how data is managed and how each stage of analysis is conducted ^64^.

## Results and dissemination

This paper outlines a protocol of a study which has not yet commenced and therefore results are not yet known. It is anticipated that recruitment of participants and running of focus groups will occur concurrently between the months of March 2021 and December 2021. Findings of the study are expected by April 2022. A summary of themes based on experiences and perceptions of physiotherapists on the role of VR in managing musculoskeletal shoulder pain will be presented and submitted for publication in a peer-reviewed journal. They will also be presented at international academic conferences in the fields of musculoskeletal and sports medicine. Anonymized data will be made available in concordance with the open data initiative and the principles of fair data management.

### Study status

The recruitment process has commenced for the current study and VR headsets have been sent to participants involved in the first focus group which is due to take place in April 2021.

## Conclusion

The current study will be the first to explore clinician’s perspectives on the role of VR in the assessment and rehabilitation of musculoskeletal shoulder pain. In addition, this study will inform the development of a VR- supported intervention for the management of shoulder pain, which will be used for further exploration of the feasibility and effectiveness of VR interventions in this population. This project is guided by a framework proposed by Birckhead *et al.* ^25^ to facilitate development of “high-quality, effective, and safe VR treatments that meaningfully improve patient outcomes”.

## Data availability

### Underlying data

No data are associated with this article.

### Extended data

Zenodo: Physiotherapist beliefs and perspectives on Virtual Reality–supported rehabilitation for the assessment and management of musculoskeletal shoulder pain: a focus group study protocol. https://doi.org/10.5281/zenodo.4633011 ^61^.

This project contains the following extended data:

- Participant information sheet and consent form,
- Question schedule,
- Researcher reflection diary NB
- Researcher reflection diary BD

Data are available under the terms of the Creative Commons Attribution 4.0 International license (CC-BY 4.0).
