# Supplementary material for: Physiotherapist beliefs and perspectives on virtual reality–supported rehabilitation for the assessment and management of musculoskeletal shoulder pain: a focus group study protocol
Source: HRB Open Res. 2022 Jan 25;4:40. Originally published 2021 Apr 27. [Version 2] doi: 10.12688/hrbopenres.13239.2 (PMC8808320; doi:10.12688/hrbopenres.13239.2)
Supplement: Peer review history

# Peer review: Reviewer response for version 2

**DOI:** 10.21956/hrbopenres.14706.r31336

**Published:** 2022-02-01

**Authors:** Thomas Matheve

**Affiliations:** 1 Spine, head and pain research group, Department of Physical Therapy and Rehabilitation, Ghent University, Ghent, Belgium; 2 Faculty of Rehabilitation Sciences, Hasselt University, Diepenbeek, Belgium

The authors addressed my questions appropriately. Good luck with your study. Looking forward to seeing the results!

Is the study design appropriate for the research question?

Yes

Is the rationale for, and objectives of, the study clearly described?

Partly

Are sufficient details of the methods provided to allow replication by others?

Partly

Are the datasets clearly presented in a useable and accessible format?

Not applicable

Reviewer Expertise:

Low back pain, rehabilitation, movement behaviour, VR

I confirm that I have read this submission and believe that I have an appropriate level of expertise to confirm that it is of an acceptable scientific standard.

# Peer review: Reviewer response for version 2

**DOI:** 10.21956/hrbopenres.14706.r31337

**Published:** 2022-01-31

**Authors:** Tasha Stanton

**Affiliations:** 1 IIMPACT in Health, University of South Australia, Adelaide, SA, Australia

The authors have responded to all of my suggested changes. Well done! I look forward to seeing the result of this study.

Is the study design appropriate for the research question?

Yes

Is the rationale for, and objectives of, the study clearly described?

Partly

Are sufficient details of the methods provided to allow replication by others?

Partly

Are the datasets clearly presented in a useable and accessible format?

Not applicable

Reviewer Expertise:

Virtual reality, pain, physiotherapy, and clinical trials. Note: I do not have expertise in qualitative analysis.

I confirm that I have read this submission and believe that I have an appropriate level of expertise to confirm that it is of an acceptable scientific standard.

# Peer review: Reviewer response for version 1

**DOI:** 10.21956/hrbopenres.14402.r29326

**Published:** 2021-05-12

**Authors:** Thomas Matheve

**Affiliations:** 1 Spine, head and pain research group, Department of Physical Therapy and Rehabilitation, Ghent University, Ghent, Belgium; 2 Faculty of Rehabilitation Sciences, Hasselt University, Diepenbeek, Belgium

The authors present a protocol for a focus group study to assess physiotherapists’ beliefs and perspectives on VR-supported assessment and rehabilitation for shoulder pain. I believe the authors should be commended for approaching the development of VR-based rehab in this methodological manner. Some aspects do need some clarification.

**Introduction**

 Overall, many positive aspects of VR are mentioned, and often references to non-musculoskeletaI literature is provided. I’m not sure whether findings in non-MSK populations can easily be extrapolated to MSK-related shoulder pain? It may be useful to refer to MSK literature where possible. If references from other patient populations are used, I believe the authors might want to be more prudent on some occasions. For example:

 Paragraph 4: The authors state: ‘VR has been proposed as a more practical and attractive option than exposure therapy *in vivo.*’ Is this also true for people with musculoskeletal pain who have movement-related fear? For example, we probably need to be able to track the movement patterns of patients and integrate this into the VR environment. This requires using movement sensors, camera systems,… So I am not sure whether, at the moment, VR is more practical for exposure therapy for movement-related fear (e.g. is it also practical for home exercises?).

 Paragraph 5: could you please provide a reference for your statement about behaviour change?

 Paragraph 6: The authors state: ‘VR has been shown as a feasible and acceptable form of rehabilitation delivery for individuals with neurological conditions.’ Quite a few studies feasibility studies for MSK rehab are available, so it may be useful to refer to some of them.

Treatment vs assessment: Although one of your study aims is to evaluate the role of VR in assessment, the introduction only refers to treatment. I believe it would be useful to also provide some background on VR assessment of MSK conditions.

**Study aims**

 Assessment and rehabilitation of MSK shoulder complaints is very broad. I wonder whether the authors have a specific type of assessment or rehab in mind (e.g., ROM exercises, strength training,…), or will this be completely dependent on the input from the physiotherapists? This is not clear at the moment. I do believe this is an important aspect to clarify. For example, in your introduction you specifically mention exposure therapy, and how VR might be useful for it. However, many MSK PTs are not familiar with this type of treatment. Do you expect that there will be sufficient input from therapists on all forms of assessment and treatment?

**Design**

 The authors state: ‘Qualitative studies provides “factual responses” to questions about how individuals perceive their health experience and how they engage with healthcare, including barriers and facilitators to healthcare use.’

 This is true, but I’m not sure this statement is applicable here as your study is about therapists’ perspectives instead of patients’ perspectives?

**Methods + Participants**

Data-saturation: It seems that data-saturation is defined as code-saturation, as you mention data saturation is reached when no new themes have emerged in the last focus group. However, code-saturation (to identify new issues/themes) is different from meaning saturation (to know exactly what is meant for each code/theme). I believe the latter is probably the aim of the study. For meaning saturation, more focus groups are probably necessary. For example, see work by Hennink *et al*. in Qual Health Res.

Participant recruitment: It is positive that the authors will try to include participants that work in different settings. Do you also consider age as an important factor to take into account? Views of older participants may differ from younger ones?

 There is a little typo at the end of the first paragraph of the recruitment section (a T is missing): he PI will then speak to potential participants…

**Procedure**

HMD: Participants will receive the Oculus Quest or Oculus Quest 2. I am not familiar with these specific types of HMDs, but as the authors stated themselves correctly, technology evolves quickly. May it be a problem that some participants will use an updated version, and hence, influence their experiences? It may be more comfortable, easier to use,… In addition, I assume the development will be done using these types of HMDs?

Games: It may be useful for the readers to have some screenshots about the VR environment of the games and of the hand controllers.

 As I understand it, participants will be asked to play a few commercially available VR games. These games are typically not designed for rehab purposes, while your aim is to develop specific VR interventions. Therefore, do you think this approach is sufficient for participants to fully understand the potential and/or barriers of VR rehab and assessment? Would it not be interesting to have participants also experience specifically designed games (potentially for other body regions) to have a better idea of these opportunities/barriers?

Timing of VR experience: Focus groups will take place between two weeks and six months of participants used the technology. This is an important issue in my opinion. There is a large difference between 2 weeks and six months. What about recall bias?

 The interview questions will explore physiotherapists perspectives on how feasible a VR intervention may be in clinical practice and anticipated barriers as well as facilitators to the use of such technology.

 This is described rather vaguely, also referring to the study aims. Could you please elaborate and provide main themes you will discuss in the focus groups? Providing the manual for the semi-structured interview may be useful.

- Will you specifically address different types of treatment/exercises? I assume participants’ views may depend on the specific aims of the exercises.
- Feasibility for home use?

Is the study design appropriate for the research question?

Yes

Is the rationale for, and objectives of, the study clearly described?

Partly

Are sufficient details of the methods provided to allow replication by others?

Partly

Are the datasets clearly presented in a useable and accessible format?

Not applicable

Reviewer Expertise:

low back pain, rehabilitation, movement behaviour, VR

I confirm that I have read this submission and believe that I have an appropriate level of expertise to confirm that it is of an acceptable scientific standard, however I have significant reservations, as outlined above.

# Response

**Published:** 2022-01-05

**Authors:** Niamh Brady

**Affiliations:** University College Cork, Ireland

Thank you for your thorough review and comments and my apologies for the delay in responding to them. Please find individual responses to your queries below:

 Introduction, Comment 1:

 Thank you – I have included more recent MSK literature in addition to literature on non-MSK populations – See Introduction, paragraphs 7-9, pages 5-6.

 Comment 2 (paragraph 4):

 Thank you for your suggestions. I have included references to two papers (Thomas *et al.*, 2016., Hennessy *et al.*, 2020) that have investigated the feasibility of using VR for graded exposure therapy in individuals with low back pain – page 4, lines 33-38. Neither of these studies examine efficacy but their results indicate that VR for this purpose is both safe and acceptable. Further research and development are required to say whether VR exposure therapy is indeed effective and whether its effect transfers to everyday activity. At present, it is uncertain whether VR exposure therapy is suitable for use at home but there is potential for it to be used in a supervised setting as shown in both studies. As stated by Hennessy *et al.*, 2020 VR offers the ability to “generate environments not otherwise possible in a clinical or laboratory setting”. As regards tracking movement, this would certainly be valuable but may not be essential depending on the goals of treatment. 

 Comment 3 (paragraph 5):

 Thank you for your comment. I have included a reference to support the change in behaviour seen after VR-based education in the study by Balsam *et al.*, 2019

 Comment 4: (paragraph 6):

 Thank you. I have revisited the literature and have included more recent and relevant research investigating VR and musculoskeletal conditions. (Intro, paragraph 7)

 Comment 5: Treatment vs Assessment:

 Thank you – this is a very important point and we have since addressed this by including some literature that has evaluated VR assessment of MSK conditions. (Intro, paragraph 9).

 Comment 6: Study Aims:

 Thank you for your comment. We intentionally left this very broad as we want to avoid influencing the participants and instead, we want to give them the opportunity to present all kinds of ideas around how VR might be used in the assessment and treatment of MSK shoulder pain. We also want the physiotherapists to think about what is relevant to them clinically and their clinical populations. For example, if we decide to focus on graded exposure and if this is not something that the clinicians themselves are familiar with or prioritise in their management then this will not reflect clinical practice for the physiotherapists included. We intend to include physiotherapists from different clinical backgrounds to facilitate an open discussion. We have now explained this more clearly in our aims.

 Comment 7: Design:

 Thank you. I agree that this may confuse the reader so I have removed this statement.

 Comment 8: Methods and Participants:

 Thank you for highlighting this and for stimulating an important discussion. We will decide on the need for further focus groups after the third focus group is complete. We plan to analyse data as it is collected so that we will begin to understand “meaning” by this point. We also expect that our data will mostly include what Hennink would describe as “concrete codes” due to the nature of the topic. This is also why we have chosen Qualitative Description as our methodology – it requires less interpretation of data collected. We are also guided by Braun and Clarke 2021 ( https://www.tandfonline.com/doi/abs/10.1080/2159676X.2019.1704846) and how they question data saturation as a useful concept in predicting sample size in qualitative research and prefer to use a more fluid approach to sample size justification.

 Comment 9: Participant Recruitment:

 Thank you. As well as clinical settings and years of experience, we will also recruit participants to represent a diverse age range. We agree that age may influence views in this situation.

 Comment 10: Typo:

 Thank you for this. It has been corrected.

 Comment 11: Procedure:

 This is a great question. The Oculus Quest and Oculus Quest 2 are both tethered devices. They are very similar. One is black and one is white. The Oculus Quest 2 is slightly lighter than the original Quest (it will be interesting to see if this is something that comes up in discussion). Participants will be advised to use software/games that are available on both devices so that the experience will be as consistent as possible.

 Yes, the software development will be done so that it can be used on Oculus HMDs.

 Comment 12: Games:

 Unfortunately, it is not possible to provide screenshots of commercial games. Instead, I have added a weblink to the Oculus website and the popular games I have referred to in the text (First Steps, Beat Saber, Sports Scramble) that is available as a demo on all devices. All participants will have the opportunity to use all of the games below.

https://www.oculus.com/experiences/quest/3675568169182204?ranking_trace=0_3675568169182204_QUESTSEARCH_904f660d-72ab-44c8-a03e-43f9830b0418

https://www.oculus.com/experiences/quest/1758986534231171?ranking_trace=0_1758986534231171_QUESTSEARCH_d0b1e295-5201-4bde-a1cb-8d2440c61b2f

https://www.oculus.com/experiences/quest/2131072803612066?ranking_trace=0_2131072803612066_QUESTSEARCH_009ba037-c88a-4b42-9363-441abbdac775

 We have intentionally left the VR experience that participants will have as open as possible. The reason is that in our opinion the software that is currently available for shoulder rehab is quite basic and software for other MSK conditions is so diverse and depends on the goal of the intervention. Therefore, we felt that this might limit the ability of clinicians to come up with their own ideas around the potential of VR in shoulder rehab. We did not want to influence the perceptions of participants by asking them to use current clinical VR software.  

 Comment 13: Interview Questions:

 Thank you. These are great suggestions. We have attached the focus group question schedule in the “Extended Data” section ( https://doi.org/10.5281/zenodo.4633011). Here you will see the suggested question schedule (which may be modified/expanded based on the first focus group interviews and emerging themes).

# Peer review: Reviewer response for version 1

**DOI:** 10.21956/hrbopenres.14402.r29325

**Published:** 2021-05-10

**Authors:** Tasha Stanton

**Affiliations:** 1 IIMPACT in Health, University of South Australia, Adelaide, SA, Australia

This protocol describes a focus group study that aims to evaluate physiotherapist beliefs and perspective on virtual reality-supported rehabilitation for the assessment and management of musculoskeletal shoulder pain. Overall, I thought this was a well written protocol and an important study for in the VR translational field. Well done to the authors! Below are my minor comments in order of appearance in the manuscript:

 Revisions:

1. Introduction, paragraph 5: The sentence, “This also led to actual or intended behaviour change in relation to management strategies”, needs a citation. Please include. Also, in the paragraph, the word “this” is used to start many of the sentences but it isn’t always clear what ‘this’ refers to. E.g., the last sentence states that “This is a feature that may be useful when working with individuals…” What is ‘this’ referring to here?
2. Introduction, final paragraph: This paragraph highlights the importance of involving both patient and provider end-users to optimize VR-based intervention design and to help identify barriers/facilitators and acceptability (e.g., Birckhead *et al’s* work). However, the Study Aims section states that the present study will only seek feedback from the providers (physiotherapists). Please provide rationale as to why providers’ input was focused on in the present study and why patient focus groups were not included. Why did you envision that the first step needed to be focus groups of physiotherapists? I am not arguing that the chosen strategy is wrong, but rather, that the rationale merely needs to be provided. This comment underpinned my choice for ‘partly’ for the rationale for the study clearly described.
3. Methods: It appears that only those physiotherapists without experience using VR will be sent a headset. Is this a problem? Given the fast pace of technology changes in the VR field (noted in the introduction), even people who say they have used immersive VR might not have used the most recent model (or the model of interest in this case). Might this create a problem if people in the focus groups do not all have the same exposure to the immersive VR equipment and games used to show the potential of an immersive platform for shoulder pain management/assessment?
4. Methods: In addition to disinfecting procedures, it might be worth considering providing a disposable VR cover to avoid direct contact with the VR headset (as this is equivalent to what you would likely need to use in a clinic given COVID-19 and may be relevant to the discussion of barriers to use).
5. Methods: Can the semi-structured interview be shared as an appendix? It is a bit difficult to follow how people’s perspectives of the general experience of VR (where none of the programs are related to shoulder pain management/assessment) will ultimately be linked to shoulder pain. This comment is what underpinned my choice of ‘partly’ for sufficient details provided to allow replication.
6. Focus group interview: Will there be consideration of the relationship status between the focus group lead and the participants (e.g., if the focus group includes physiotherapists that were previously students of any of the researchers)? Given that feedback/discussion within the focus groups is not anonymous, such previous relationships may feasibly influence the nature/outcome of focus group discussion.

Is the study design appropriate for the research question?

Yes

Is the rationale for, and objectives of, the study clearly described?

Partly

Are sufficient details of the methods provided to allow replication by others?

Partly

Are the datasets clearly presented in a useable and accessible format?

Not applicable

Reviewer Expertise:

Virtual reality, pain, physiotherapy, and clinical trials. Note: I do not have expertise in qualitative analysis.

I confirm that I have read this submission and believe that I have an appropriate level of expertise to confirm that it is of an acceptable scientific standard, however I have significant reservations, as outlined above.

# Response

**Published:** 2022-01-05

**Authors:** Niamh Brady

**Affiliations:** University College Cork, Ireland

Thank you kindly for your very helpful comments and my apologies for the delay in responding. I have addressed and responded to your comments to the best of my ability below:

 Comment 1:

 Thank you. I have reworded the paragraph to make it clear that I am referring to VR-based education and I have included a reference to support the change in behaviour seen after VR-based education in the study by Balsam *et al.*, 2019 (see "Introduction" paragraph 5, page 4).

 Comment 2:

 This is an excellent point. In our original discussions, we had suggested doing two separate focus group studies, one including physiotherapists and another including patients. In the end, we felt that a focus group would be a more suitable environment for physiotherapists to share ideas about a novel intervention, but that patients may find this difficult, particularly when talking about something that has not yet been developed. We do still plan to include patients in a qualitative study, but this will come later, and we will conduct one-to-one interviews rather than focus group interviews. Before carrying out these one-to-one interviews, patients will have had the chance to use VR technology designed for managing shoulder pain in a supervised setting. This is not feasible at present due to COVID-19 restrictions on research activities in Ireland. I have added a sentence in the introduction to state that a future study including patients will be carried out.

 Comment 3:

 This is a great question. Our rationale here was to keep the VR experience that participants have as open as possible so as not to influence perceptions and ideas. We felt that experience of different types of VR (hardware and software) would lead to a more open discussion and introduction of ideas from different points of view. We anticipated that there would be a mix of volunteers with/without prior VR experience when in fact, most volunteers so far have no prior VR experience and those that have, have used the Oculus Quest. In addition, we did not specify what game participants should play in the trial period for the very same reason.

 Comment 4:

 Thank you for this suggestion – we will take this on board and aim to implement same.

 Comment 5:

 Yes of course. The question schedule is already attached in the "Extended Data” section ( https://doi.org/10.5281/zenodo.4633011). 

 Comment 6:

 Thank you for making this point. We will not include clinicians who have relationships (family, colleagues, and students) to the primary investigator or facilitator in the focus group. This will be made clear in our methods section. (page 9 – lines 29-30). We will, however, ask a colleague to participate in a pilot interview to gain feedback on the questions schedule. This individual will not be included in the focus group itself due to the reasons you have pointed out.
